# Aerobic exercise training prevents impairment in renal parameters and in body composition of rats fed a high sucrose diet

**DOI:** 10.1186/s13104-021-05790-7

**Published:** 2021-09-26

**Authors:** Jaqueline A. de Souza, Angélica B. Gonçalves Pinto, Emerson C. de Oliveira, Daniel B. Coelho, Nádia L. Totou, Wanderson G. de Lima, Lenice K. Becker

**Affiliations:** 1 Physical Education School, Federal University of Ouro Preto (UFOP), Ouro Preto, MG 35,400-000 Brazil; 2 Dept. of Biological Sciences-ICEB, UFOP, Ouro Preto, MG Brazil; 3 Dept. of Pharmacology, ICB, Federal University of Minas Gerais, Belo Horizonte, MG Brazil

**Keywords:** SUD, Swimming, Renal function, Body composition

## Abstract

**Objective:**

This study aimed to evaluate the effect of swimming training (T) on the renal system and body composition parameters in young animals treated with a high sucrose diet (SUD) during 12 weeks.

**Results:**

The SUD impaired the physical performance, increased the body adiposity index (BAI), Lee index (LI) and retroperitoneal adipose tissue (RAT) weight, plasma creatinine and number renal cells nuclei, decreased urinary volume and urinary creatinine excretion besides creatinine clearance. The T reversed the increased the BAI, LI, RAT weight, plasma and urinary creatinine, creatinine clearance and number renal cells nuclei in addition to promoting decrease in urinary protein excretion. This study found that eight weeks of swimming physical training protected renal function and restored normal glomerular filtration rate (GFR) values. Swimming training also contributed to prevention of the onset of a renal inflammatory process and caused a decrease in the risk of development of obesity promoted by SUD decreasing the body composition parameters (BAI, LI, and RAT weight).

**Supplementary Information:**

The online version contains supplementary material available at 10.1186/s13104-021-05790-7.

## Introduction

A high sugar level is a factor that has been linked to an increase in kidney disease and association with some risk factors, including diabetes, gout, metabolic syndrome [1–4] and the development of obesity. Besides an increase in oxidative stress and nitric oxide inactivation via reactive oxygen species, high sugar also induces high level of creatinine and blood urea nitrogen [5]. Sucrose, specifically, can promote an increase in proteinuria and a higher percentage of abnormal glomeruli in obese rats [6].

Physical exercise is recognized to promote alterations in renal parameters in experimental animal models [7–9] those alterations provide renal protective effects. The exercise training neutralize the accumulation of renal triglycerides associated with high sucrose diet, which is partially mediated by upregulation of fatty acid β-oxidation [7].

Data have shown that a metabolic re-programming process subsequent to early-life insults, i.e. maternal life or childhood can take to development of chronic diseases [10, 11]. The use of food rich in refined sugar has been shown to exponentially raise glucose blood levels, increase the total caloric intake and the prevalence of metabolic syndrome in children and adolescents these populations [12].

A balanced diet and regular physical exercise are of great relevance in preventing obesity, cardiovascular and kidney diseases, justifying the development of studies that assess the beneficial effects of regular physical training in childhood that will contribute to the decrease in deleterious effects of consuming a diet rich in simple carbohydrates.

Thus, this study aimed to evaluate the effect of swimming training (T) on the renal system and body composition parameters [body adiposity and Lee indices (BAI and LI, respectively) and retroperitoneal adipose tissue (RAT) weight] in animals treated with SUD.

## Main text

### Methods

#### Animals

38 male Wistar rats, age 21 days (50 g) (come from the CCA-UFOP) divided randomly, into 4 groups (4 animals/box): (1) sedentary rats fed with standard diet (S-SD), n = 14; (2) trained rats fed with standard diet (T-SD), n = 9; (3) sedentary rats fed with high sucrose diet (S-SUD), n = 05; and (4) trained rats fed with SUD (T-SUD), n = 10; marked on the tail; allocated in a room at a temperature of 24 ± 2 °C and 12-h light/dark cycle (7 a.m.–7 p.m.). *Body weight assessments:* realized at weeks 1, 4, 5, 7, and 11. *Food intake assessments:* realized at weeks 1, 5, and 10. *Approval of the study:* by the Ethics Committee for Animal Use, UFOP (Protocol 45/2014). The experimental design scheme was shown in Additional file 2: Figure S2.

#### Diets

SD—commercial rodent feed in pellets (Nuvilab CR1 Quimtia®) was administered for 12 weeks from weaning and SUD as previously published [13] by 8 weeks. After 4 weeks initial was started the exercise training program.

#### Exercise training and evaluation of endurance capacity

*Local—*collective glass tank of CCA-UFOP with warm water at 28 ± 2 °C and 45 cm depth. *Adaptation period:* 15 min (first day) increased by 15 min each day until reaching 60 min (fourth day) (*adapted from* [14]). *Maximal test:* at the 4th (started training) and 12th (24 h after the last training section) weeks as proposed by others authors [15]. Fatigue point was *adapted from* [16]*. Training:* swimming for 8 weeks. The first 4 weeks didn’t use a workload, but that was added at the fifth week (60% of that obtained in maximal test) as *adapted from* [15].

#### Water intake, urinary volume and water balance measurements in 24 h

Rats were allocated in metabolic cages (Tecnoplast® SPA) during the 12-week period (CCA-UFOP); weighed (SF–400 scale), individually housed for a period of 24 h with free access to water and food. Measurements of urine volume and water intake were realized. Samples of urine 1.5 mL were obtained, labeled, and frozen at − 20 °C. Calculation of water balance was done according to the equation:

1 $$Water\;Balance\left( {mL/24h} \right) = (Water\;Intake\left[ {mL/24h} \right] - Urinary\;Volume\left[ {mL/24h} \right]).$$

#### Euthanasia

48 h after the last T session. *Collected materials:* Adipose tissues inguinal, retroperitoneal and epididymal (IAT, RAT and EAT, respectively), blood and kidneys. Blood was centrifuged (CENTRIBIO 80-2 B centrifuge) at 3000 rpm for 10 min to separate the plasma and then maintained at a temperature of − 20 °C. Details: Additional file 4: Table S2.

#### Determination of the LI

LI was measured according to [17]. Animals’ body mass and the nasoanal length were measured [17]. It was used formula below:

2 $$\frac{{\sqrt[3]{BM \left( g \right)}}}{{NL \left( {cm} \right)}}$$

#### Determination of the BAI

BAI was measured according to [18]. The EAT, IAT, and RAT were removed and weighed (BEL precision scale), and used in the following equation:

3 $$\frac{EAT \times IAT \times RAT\left( g \right)}{{BW \left( g \right)}} \times 100$$

#### Evaluation of RAT

RAT analyse provide an assessment of the risk of developing cardiovascular diseases [19].

#### Plasma and Urine Creatinine Concentration and Urine Protein

*Samples—*plasma and urine; *kit—*commercially available kit (Labtest, Belo Horizonte/MG, Brazil)—by colorimetric modified Jaffé approach. Calculation of creatinine clearance (CrCl):

4 $$\frac{{CrCl \left( {mL/min} \right) = \left[ {Urine\;Creatinine\left( {mg/dL} \right) \times Urine\;Volume\left( {mL/24h} \right)} \right]}}{{\left[ {Serum\;Creatinine\left( {mg/dL} \right) \times 1440\left( {min} \right)} \right]}}$$

Proteinuria was determined with the use of pyrogallol red technique using a commercially available kit (Bioclin, Belo Horizonte, Brazil), *n* = 38 rats. Results were expressed in mg/mg creatinine.

#### Renal histology

*Collected material—*right kidneys; *Stage and calculation—*as described by others [20, 21]. *Equipment—*light microscope (Leica DM5000). *Analysis—*Analysis and Image Processing Software Leica Qwin (Germany) [22].

#### Data and statistical analysis

*Normality test—*Kolmogorov–Smirnov. *Statistical tests used—*One-way or two-way analysis of variance (ANOVA) and Tukey’s post-test for multiple comparisons following ANOVA. *Software used—*GraphPad Prism 7.0 for Windows (GraphPad Software, San Diego California USA). Data were expressed as mean ± standard deviation of mean and differences between pairs of means were considered significant when the probability of type I error was less than 5% (p < 0.05). The data were analyzed blindly by the researchers involved.

### Results

#### Confirmation of the effectiveness of the exercise training employed

SUD affected the physical performance at the 12nd week since only the T-SD group had a longer exhaustion time with an increase of 16.9% (Table 1).

**Table 1:** Exaustion times and hydrics parameters for SD and SUD rats, sedentary and trained

	Standard diet (SD)		Sucrose diet (SUD)	
	Sedentary (S-SD)	Trained (T-SD)	Sedentary (S-SUD)	Trained (T-SUD)
Initial ET (s)	720 ± 44 (n = 04)	774 ± 73 (n = 09)	819 ± 74 (n = 05)	770 ± 74 (n = 10)
Final ET (s)	709 ± 53 (n = 04)	853 ± 92* (n = 09)	791 ± 47 (n = 05)	782 ± 73 (n = 10)
Water intake (mL/24 h)	29.5 ± 5.7 (n = 14)	33.9 ± 4.2 (n = 09)	29.0 ± 2.2 (n = 05)	30.9 ± 1.9 (n = 10)
Urinary volume (mL/24 h)	14 ± 2.4 (n = 14)	9 ± 3.9 (n = 09)	8 ± 1.4* (n = 05)	14 ± 5.9 (n = 10)
Water balance (mL/24 h)	15.8 ± 5.9 (n = 14)	24.6 ± 6.4* (n = 09)	21.1 ± 3.5 (n = 05)	17.3 ± 5.5 + (n = 10)

Exaustion times before (Initial ET) and after (Final ET) were realized on the 4th and 12nd (24 h after the last training section) weeks. It was used the parametric test, two-way ANOVA followed by Tukey’s post-test; p < 0.05. *different from S-SD group. Measurements of water intake and urinary volume were realized on the metabolic cages, after 24 h. It was used the parametric test, two-way ANOVA followed by Tukey’s post-test; p < 0.05. ^*^different of S-SD group; ^+^different of T-SD group

#### Effects of the exercise training on body composition

At the 11th week, SUD caused a decrease of 23.8% in the body weight gain and an increase in the BAI (69%), LI (10.5%) and RAT weight (28.6%) as shown in Additional file 1: Figure S1, panels A to C. T caused a decrease of 15.2% in body weight gain only in the T-SD (Additional file 3: Table S1) and a decrease in BAI (81%), LI (15.8%), and RAT weight (55.1%) in T-SUD, decreasing only the BAI (54.9%) and RAT weight (26.5%) in T-SD.

#### Effects of the exercise training on 24-h parameters hydric

SUD caused a decrease of 42.9% the urinary volume, while and T restored these values to normal levels (Table 1). However, T caused an increase of 36% in water balance in the T-SD (Table 1).

#### Effects of the exercise training on the renal biochemical parameters

**Fig. 1:**
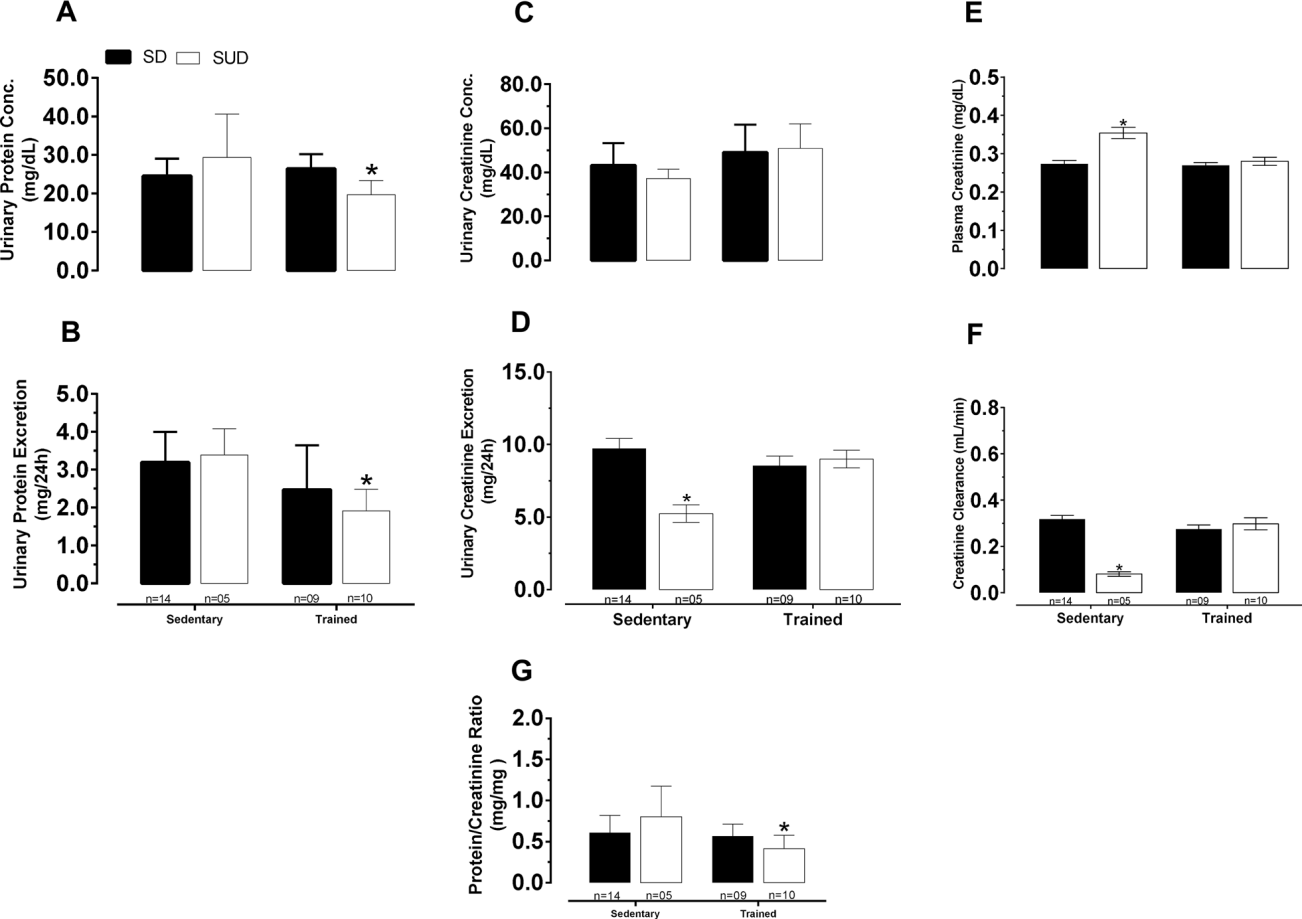
Urinary protein concentration values (**A**), *different from S-SUD group. Urinary protein excretion values (**B**), *different from S-SD and S-SUD groups. Urinary creatinine concentration values (**C**), there was no difference between groups. Urinary creatinine excretion values (**D**), *different from S-SD, T-SD and T-SUD groups. Plasma creatinine (**E**), *different from S-SD, T-SD and T-SUD groups. Creatinine clearance values (**F**), *different from S-SD, T-SD and T-SUD groups. Protein/Creatinine ratio values (**G**), *different from S-SUD group. SD (Standard Diet), SUD (Sucrose Diet) and T (swimming training). All measurements were realized after 8th week of T. It was used the parametric test, two-way ANOVA followed by Tukey’s post-test; p < 0.05. The bars represent group mean data. Differences among the pairs of means are indicated by different signs

SUD increased in 22.9% the plasma creatinine (Fig. 1E) and decreased in 53.8% and 75% the urinary creatinine excretion and creatinine clearance, respectively (Fig. 1D, F, respectively). T protected the kidney against SUD-induced modifications of plasma and urinary creatinine, creatinine clearance besides decreased urinary protein concentration and excretion in addition to protein/creatinine ratio.

#### Effects of the exercise training on renal structure

**Fig. 2:**
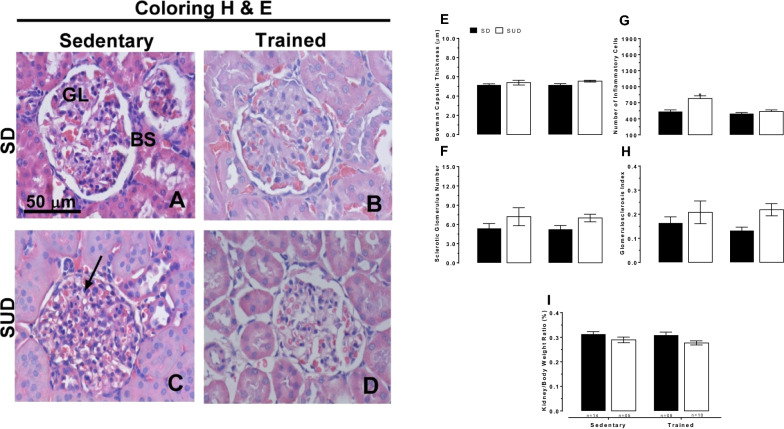
Histological sections stained with hematoxylin and eosin (**A** to **D**). Bowman Capsule Thickness (**E**), there was no difference among the groups. Sclerotic glomerulus number (**F**), there was no difference among the groups. Number of inflammatory cells values (**G**), *different from S-SD, T-SD and T-SUD. Glomerulosclerosis index (**H**), there was no difference among the groups. Kidney/body weight ratio values (**I**), there was no difference among the groups. All measurements were realized after 8th week of T. *GL* glomeruli, *BS* Bowman Space, *SD* Standart Diet, *SUD* Sucrose Diet and *T* swimming training. It was used the parametric test, two-way ANOVA followed by Tukey’s post-test; p < 0.05. The bars represent group mean data. Differences among the pairs of means are indicated by different signs

SUD increased in 31.5% in cell nuclei (Fig. 2C, third quadrant left side, and G) and T reduced in 31.1% about this parameter.

### Discussion

This study found that eight weeks of swimming physical training protected renal function and restored normal GFR values. Swimming training also contributed to prevention of the onset of a renal inflammatory process and decrease the risk of development of obesity induced by SUD since the body composition parameters (BAI, LI, and RAT weight) were lower in trained group.

Although some authors have not observed impairment in the physical performance of animals fed a diet rich in simple carbohydrates [23], SUD contributed negatively to the physical performance of the animals in our study. After 12 weeks of swimming exercise training, the total time to exhaustion was higher only in animals of the T-SD group.

Sugar seems to increase parameters, such as BAI [18, 24–26], LI [26], and RAT weight [27, 28] that contribute to the development of cardiovascular diseases [25]. In our study we observed that all these parameters were increased by SUD. Our results show that the exercise reduced BAI in the T-SUD (81%) and T-SD (54.9%) groups and LI and RAT weight in the T-SUD group although some studies haven’t shown the effects of physical exercise in reducing the BAI, LI and RAT weight [13, 19, 24, 29] promoted by high sugar content. A possible explanation for these exercise-associated effects is energetic homeostasis tends to been maintained in mammals through a balance between energy intake and expenditure, thus influencing body weight [30] doing the T have an important role in controlling these factor [31, 32].

Some studies have shown that a sugar intake can have influence (or not) in parameters like water intake [8, 33, 34], urinary volume [8], and water balance [8] in rodents. In this study, SUD didn’t affect water intake in any group and decreased urinary volume, but the exercise training restored to normal levels. The T-SD group had a higher water balance, demonstrating that swimming promotes changes in the hydric balance depending on the diet to which it was assigned. The T-SD group seems to have had a higher expenditure of water by other means than urinary excretion. Therefore, when swimming was undertaken by the SUD group, these differences don’t appear. There are no studies that demonstrate how changes in water balance occur in response to sugar overload in rodents and outline the mechanisms by which this process happens. Studies that investigate the hydric parameters are necessary to clarify many of the physiological changes that may occur in rodents fed with sugar overload, since these issues may be related to some compensation of the physiological system to maintain homeostasis, mainly of the renal system.

It is known that the overload sugar can modifying the following parameters: (1) proteinuria [34, 35], (2) plasma and urinary creatinine [34], and (3) creatinine clearance (used as estimate of the GFR) [34, 36]. In our study, SUD increased plasma creatinine and decreased urinary creatinine excretion and creatinine clearance. T led to a reversal in GFR alterations by bringing this parameter back to normal levels corroborating with others [7]. In addition, the T decreased urinary protein concentration and excretion and in protein/creatinine ratio.

Sugar has also been seen as a villain that can cause renal inflammation, as noted in other study [37]. In our research, SUD caused an increase in cell nuclei suggesting an increase in the number inflammatory infiltrates. T protected against these SUD-induced changes (decreasing this parameter in 31.1%), thus corroborating the results presented by others authors [38]. All of the other parameters weren’t affected neither by SUD nor for T (Fig. 2E, F, H, and I).

It was observed that damage to kidney function appeared earlier than structural renal damage in response to excess sugar in the SUD. High glucose-induced oncotic pressure alterations didn’t initially modify the structure of the glomerulus but did modify renal function probably by increasing the excretion of urinary proteins, thus increasing proteinuria and decreasing GRF [39]. In addition, the time of exposure to the diet and age of animals (young animals) could have provided protection for the renal structure conferred by mechanisms of adaptation to sugar overload.

Collectively, our study demonstrated that T prevented SUD-induced impairment of kidney functional parameters and was efficient in preventing an SUD-induced increase adipose tissue deposits.

## Limitations

We lacked investigate the cardiovascular parameters complementing the data aiming to observe the effects of sugar overload also on these parameters, being able to make a direct relationship between renal parameters, body composition and cardiovascular parameters in response to SUD and T.

## Supplementary Information

**Additional file 1: Figure S1.** Body adiposity index values (panel A), *different from S-SD, T-SD and T-SUD groups, ^+^different from S-SD group. Lee index values (panel B), *different from S-SD, T-SD, T-SUD groups. Retroperitoneal adipose tissue weight values (panel C), *different from S-SD, T-SD and T-SUD groups, **different from S-SD group. It was used the parametric test, two-way ANOVA followed by Tukey’s post-test; p < 0.05; all the measurements above were realized after the 8^th^ week of T. SD (Standart Diet), SUD (Sucrose Diet) and T (swimming training). The bars represent group mean data. Differences among the pairs of means are indicated by different signs.

**Additional file 2: Figure S2.** Scheme of experimental design showing the different stages for the development of the model, allocation of animals in different groups and main experimental tests carried out in this study. Weaned rats from CCA-UFOP were fed diets containing 68% carbohydrates, 33% standard commercial feed, 33% condensed milk, 7% sucrose and the remaining water or standard pellet diet for 12 weeks since weaning. In the 4th week, the rats were adapted (4 days) to the aquatic environment and the animals related to the trained groups underwent swimming training for 8 weeks. The exhaustion time protocol was performed at weeks 4 and 12 to assess the animals' physical performance. The measurements of water intake and urinary volume performed in a metabolic cage were performed on the 12nd week, in addition to the renal (for analysis of renal function and histology) and body composition (BAI, LI and RAT weight) experiments, which were performed 48 h after last T session at the end of the 12nd week, at which time the rats were euthanized and samples of blood and kidney tissue were collected for analysis. Body weight measurements were taken at weeks 1, 4, 5, 7 and 11 and food intake measurements were taken at weeks 1, 5 and 10. CCA-UFOP (Center of Animal Science of UFOP); SD (standart diet); SUD (sucrose diet); T (swimming training).

**Additional file 3: Table S1.** Body weight measurements and food intake before and after T.

**Additional file 4: Table S2.** Table with additional information about the study.

## Data Availability

The datasets used and/or analyzed during the current study are available from the corresponding author on reasonable request.

## Abbreviations

UFOP: Federal University of Ouro Preto
T: Swimming training
SUD: High sucrose diet
S-SD: Sedentary standard diet
S-SUD: Sedentary SUD
T-SD: Trained standard diet
T-SUD: Trained SUD
BAI: Body adiposity index
LI: Lee index
RAT: Retroperitoneal adipose tissue
EAT: Epididymal adipose tissue
IAT: Inguinal adipose tissue
RAT: Retroperitoneal adipose tissue
CrCl: Creatinine clearance
GFR: Glomerular filtration rate
CCA-UFOP: Center of Animal Science of UFOP
