# Supplementary material for: Aerobic exercise training prevents impairment in renal parameters and in body composition of rats fed a high sucrose diet
Source: BMC Res Notes. 2021 Sep 26;14:378. doi: 10.1186/s13104-021-05790-7 (PMC8474763; doi:10.1186/s13104-021-05790-7)
Supplement: Supplementary file 1 — Additional file 1: Figure S1. Body adiposity index values (panel A), *different from S-SD, T-SD and T-SUD groups, +different from S-SD group. Lee index values (panel B), *different from S-SD, T-SD, T-SUD groups. Retroperitoneal adipose tissue weight values (panel C), *different from S-SD, T-SD and T-SUD groups, **different from S-SD group. It was used the parametric test, two-way ANOVA followed by Tukey’s post-test; p < 0.05; all the measurements above were realized after the 8th week of T. SD (Standart Diet), SUD (Sucrose Diet) and T (swimming training). The bars represent group mean data. Differences among the pairs of means are indicated by different signs. [file 13104_2021_5790_MOESM1_ESM.docx]

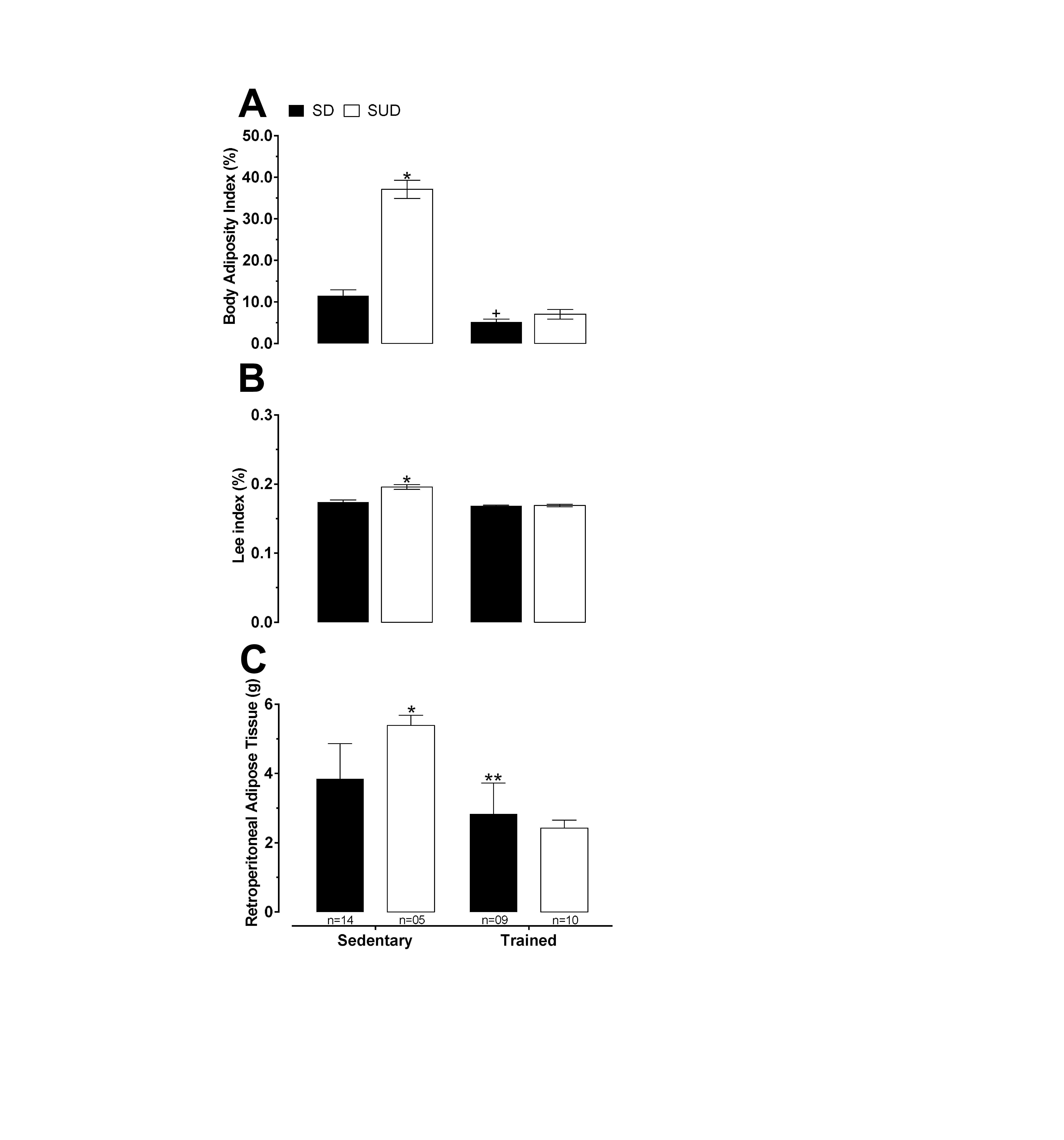


**Figure S1:** Body adiposity index values (panel A), *different from S-SD, T-SD and T-SUD groups, ^+^different from S-SD group. Lee index values (panel B), *different from S-SD, T-SD, T-SUD groups. Retroperitoneal adipose tissue weight values (panel C), *different from S-SD, T-SD and T-SUD groups, **different from S-SD group. It was used the parametric test, two-way ANOVA followed by Tukey’s post-test; p<0.05; all the measurements above were realized after the 8^th^ week of T. SD (Standart Diet), SUD (Sucrose Diet) and T (swimming training). The bars represent group mean data. Differences among the pairs of means are indicated by different signs.
