# Supplementary material for: Aerobic exercise training prevents impairment in renal parameters and in body composition of rats fed a high sucrose diet
Source: BMC Res Notes. 2021 Sep 26;14:378. doi: 10.1186/s13104-021-05790-7 (PMC8474763; doi:10.1186/s13104-021-05790-7)
Supplement: Supplementary file 2 — Additional file 2: Figure S2. Scheme of experimental design showing the different stages for the development of the model, allocation of animals in different groups and main experimental tests carried out in this study. Weaned rats from CCA-UFOP were fed diets containing 68% carbohydrates, 33% standard commercial feed, 33% condensed milk, 7% sucrose and the remaining water or standard pellet diet for 12 weeks since weaning. In the 4th week, the rats were adapted (4 days) to the aquatic environment and the animals related to the trained groups underwent swimming training for 8 weeks. The exhaustion time protocol was performed at weeks 4 and 12 to assess the animals' physical performance. The measurements of water intake and urinary volume performed in a metabolic cage were performed on the 12nd week, in addition to the renal (for analysis of renal function and histology) and body composition (BAI, LI and RAT weight) experiments, which were performed 48 h after last T session at the end of the 12nd week, at which time the rats were euthanized and samples of blood and kidney tissue were collected for analysis. Body weight measurements were taken at weeks 1, 4, 5, 7 and 11 and food intake measurements were taken at weeks 1, 5 and 10. CCA-UFOP (Center of Animal Science of UFOP); SD (standart diet); SUD (sucrose diet); T (swimming training). [file 13104_2021_5790_MOESM2_ESM.docx]

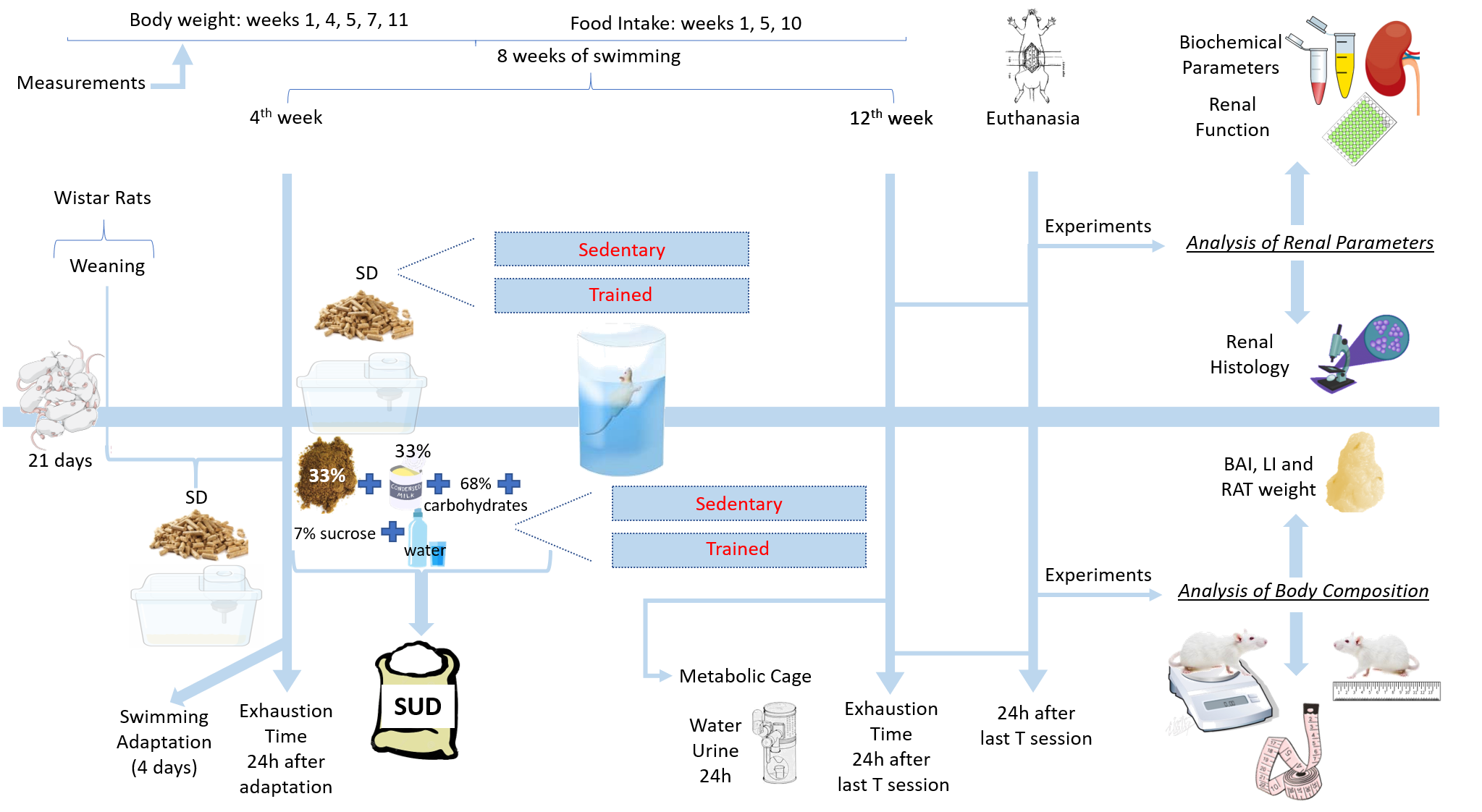


**Figure S2:** Scheme of experimental design showing the different stages for the development of the model, allocation of animals in different groups and main experimental tests carried out in this study. Weaned rats from CCA-UFOP were fed diets containing 68% carbohydrates, 33% standard commercial feed, 33% condensed milk, 7% sucrose and the remaining water or standard pellet diet for 12 weeks since weaning. In the 4^th^ week, the rats were adapted (4 days) to the aquatic environment and the animals related to the trained groups underwent swimming training for 8 weeks. The exhaustion time protocol was performed at weeks 4 and 12 to assess the animals' physical performance. The measurements of water intake and urinary volume performed in a metabolic cage were performed on the 12^nd^ week, in addition to the renal (for analysis of renal function and histology) and body composition (BAI, LI and RAT weight) experiments, which were performed 48 hours after last T session at the end of the 12^nd^ week, at which time the rats were euthanized and samples of blood and kidney tissue were collected for analysis. Body weight measurements were taken at weeks 1, 4, 5, 7 and 11 and food intake measurements were taken at weeks 1, 5 and 10. CCA-UFOP (Center of Animal Science of UFOP); SD (standart diet); SUD (sucrose diet); T (swimming training).
