# Supplementary material for: Aerobic exercise training prevents impairment in renal parameters and in body composition of rats fed a high sucrose diet
Source: BMC Res Notes. 2021 Sep 26;14:378. doi: 10.1186/s13104-021-05790-7 (PMC8474763; doi:10.1186/s13104-021-05790-7)
Supplement: Supplementary file 3 — Additional file 3: Table S1. Body weight measurements and food intake before and after T. [file 13104_2021_5790_MOESM3_ESM.docx]

**Table S1:** Body weight measurements and food intake before and after T.

| VARIABLE 1 | Before T (4 weeks under diet) | |  | | After T (12 weeks under diet) | | | | |
| --- | --- | --- | --- | --- | --- | --- | --- | --- | --- |
|  | ***Standard Diet (SD)*** | ***Sucrose Diet (SUD)*** | |  | ***Standard Diet***  ***(SD)*** | |  | ***Sucrose Diet***  ***(SUD)*** | |
|  |  |  |  |  | ***Sedentary***  ***(S-SD)*** | ***Trained***  ***(T-SD)*** |  | ***Sedentary***  ***(S-SUD)*** | ***Trained***  ***(T-SUD)*** |
| Body Weight (g) | 207±54  (n=23) | 161±19^*^  (n=15) | |  | 441±31  (n=14) | 374±27^+^  (n=09) |  | 336±25^+^  (n=05) | 345±38^+^  (n=10) |
| VARIABLE 2 | **Mean of the 3 measures of the groups** | | | | | | | | |
|  | **_** | **_** | |  | ***Sedentary***  ***(S-SD)*** | ***Trained***  ***(T-SD)*** |  | ***Sedentary***  ***(S-SUD)*** | ***Trained***  ***(T-SUD)*** |
| Food Intake (g) | **_** | **_** | |  | 15.0±4.5  (n=14) | 17.7±4.5  (n=09) |  | 14.0±2.2  (n=05) | 10.0±1.9  (n=10) |

Body weight measurements were realized in five moments - week 1, week 4, week 5, week 7, week 11. It was used the parametric test, two-way ANOVA followed by Tukey’s post-test; p<0.05. ^*^different from SD groups; ^+^different from S-SD;

Food intake values were realized in three moments – week 1, week 5, week 10, there was no difference among the groups. It was used the parametric test, one-way ANOVA followed by Tukey’s post-test; p<0.05.
