# Supplementary material for: Aerobic exercise training prevents impairment in renal parameters and in body composition of rats fed a high sucrose diet
Source: BMC Res Notes. 2021 Sep 26;14:378. doi: 10.1186/s13104-021-05790-7 (PMC8474763; doi:10.1186/s13104-021-05790-7)
Supplement: Supplementary file 4 — Additional file 4: Table S2. Table with additional information about the study. [file 13104_2021_5790_MOESM4_ESM.docx]

**Table S2:** Table with additional information about the study.

| ADDITIONAL STUDY INFORMATION | |
| --- | --- |
| Criteria for the inclusion of animals in the study | Animals with normal growth conditions and healthy aspect; |
| Criteria for the exclusion of animals in the study | Animals without normal growth conditions and healthy aspect; |
| Animals excluded of the study | Five animals were excluded of the study, for not presenting themselves within the aspects pertinent to the inclusion criteria in the study; |
| Handling of the animals | This action was carried out with great care to minimize stress. The animals were handled only on the back, following the guidelines proposed by the Ethics Committee on Animal Research at UFOP. |
| Monitoring of the animals | This monitoring was realized throughout the experimental protocol at different times of the day, taking into account the verification of the temperature of the environment in which they were, adequate lighting conditions, verification of possible noises that could stress the animals, as well as periods of adaptation and acclimatization to the environment, taking them 1 hour before the place that would be carried out experiments to adapt to the new condition. |
| Number of animals per experimental procedures | 1. Exercise training and evaluation of endurance capacity: *n* = 28 rats; 2. Water intake, urinary volume and water balance measurements in 24h: *n* = 38 rats; 3. Determination of the LI: *n* = 38 rats; 4. Determination of the BAI: *n* = 38 rats; 5. Plasma and urine creatinine concentration and urine protein: *n* = 38 rats; 6. Renal histology: *n* = 38 rats |
| Note | The experiments listed below were carried out at the Exercise Physiology and Morphopathology laboratories at UFOP;  Euthanasia; Determination of the LI; Determination of the BAI; Plasma and Urine Creatinine Concentration and Urine Protein; Renal Histology; |
| Justification of the choice of experimental procedures | The experimental procedures were based on what is already well established in the literature to evaluate the parameters chosen for investigation in this study. |
| Sample calculation | This calculation was realized using the BioStat 2008 5.0.1 software, taking into account the main study variables (renal parameters). |
| Euthanasia Methods | The death was carried out with an overdose of anesthetic, ketamine (300 mg/kg, ip) plus xylazine (30 mg/kg, ip), as proposed by the UNIFESP's Guide to Anesthesia and Analgesia in Laboratory Animals (2019), in which demonstrates that a dosage of anesthetic is necessary for the euthanasia of rats, comprising about 3 times the value of the normal dose. This simplified euthanasia protocol was chosen since the analyzes to be performed did not require a more complex euthanasia strategy. |
